# Food Allergy Prevalence in Salvadoran Schoolchildren Estimated by Parent-Report

**DOI:** 10.3390/ijerph15112446

**Published:** 2018-11-02

**Authors:** Francisco Cabrera-Chávez, Cecilia Ivonne Rodríguez-Bellegarrigue, Oscar Gerardo Figueroa-Salcido, Jesús Aristeo Lopez-Gallardo, Jesús Gilberto Arámburo-Gálvez, Marcela de Jesús Vergara-Jiménez, Mónica Lizzette Castro-Acosta, Norberto Sotelo-Cruz, Martina Hilda Gracia-Valenzuela, Noé Ontiveros

**Affiliations:** 1 Nutrition Sciences Academic Unit, Autonomous University of Sinaloa, Cedros y Calle Sauces S/N, Fracc. Los Fresnos, Culiacán 80019, Sinaloa, Mexico; fcabrera@uas.edu.mx (F.C.-C.); gerardofs95@hotmail.com (O.G.F.-S.); aristeo.lopez37@hotmail.com (J.A.L.-G.); mjvergara@uas.edu.mx (M.d.J.V.-J.); cmonica@uas.edu.mx (M.L.C.-A.); 2 Luis Edmundo Vasquez School of Health Sciences, Department of Public Health, Dr. José Matías Delgado University, Antiguo Cuscatlán 1502, El Salvador; cirodriguezb@ujmd.edu.sv; 3 Department of Biological Chemistry Sciences, University of Sonora, Hermosillo 83000, Sonora, Mexico; gilberto.aramburo.g@gmail.com; 4 Department of Medicine and Health Sciences, University of Sonora, Hermosillo 83000, Sonora, Mexico; nsotelo51@gmail.com; 5 Technological Institute of the Yaqui Valley, Bácum 82276, Valle del Yaqui, Sonora, Mexico; 6 Division of Sciences and Engineering, Department of Chemical, Biological, and Agricultural Sciences (DC-QB), University of Sonora, Navojoa 85880, Sonora, Mexico

**Keywords:** food allergy, prevalence, parent-reported, schoolchildren, anaphylaxis

## Abstract

The prevalence of food allergy (FA) has not been estimated at a population level in Central American countries and, consequently, the magnitude and relevance of the problem in the Central American region remains unknown. Thus, our aim was to evaluate the parent-reported prevalence of FA in a population of schoolchildren from the Central American country El Salvador. A Spanish version of a structured questionnaire was utilized. Five hundred and eight (508) parents returned the questionnaire with valid responses (response rate, 32%). The estimated prevalence rates (95% CI) were: adverse food reactions 15.9 (13.0–19.3), “perceived FA, ever” 11.6 (9.1–14.6), “physician-diagnosed FA, ever” 5.7% (4.0–8.0), “immediate-type FA, ever” 8.8% (6.6–11.6), “immediate-type FA, current” 5.3% (3.6–7.6), and anaphylaxis 2.5% (1.5–4.3). The most common food allergens were milk (1.7%), shrimp (1.3), chili (0.7%), chocolate (0.7%), and nuts (0.3%). Most of the “food-dependent anaphylaxis” cases (60.5%) sought medical attention, but only one case reported the prescription of an epinephrine autoinjector. Mild and severe FA cases are not uncommon among Salvadoran schoolchildren and both the prescription of epinephrine autoinjectors by healthcare personnel and the use of the autoinjectors by anaphylactic individuals should be encouraged.

## 1. Introduction

Food allergy (FA) is an immune disorder triggered by the ingestion of the relevant allergenic food and its symptoms are specific and reproducible [1,2]. The condition negatively impacts on socioeconomic aspects and it is associated with a low quality of life [3,4,5]. Furthermore, allergic individuals or their parents should be trained to properly interpret food labels in order to avoid accidental exposures to the allergen of interest [6]. Notably, FA cases seems to be increasing in both high income and developing countries and it has been estimated that affects 6–8% and 2–4% of children and adults, respectively [7]. However, the prevalence of FA and the main allergenic foods implicated varies not only among different age groups but also geographically [8,9]. Certainly, the prevalence of FA is well documented in most high income countries [10,11,12], but just a few population-based studies have been carried out in order to evaluate the prevalence of FA in the geographical area of Latin America [1,2,13,14]. Particularly, there is a lack of information about the prevalence of FA at population level in the Central American region. Thus, our aim was to conduct a survey-based cross-sectional study in order to estimate the prevalence of FA in school-aged children from San Salvador, El Salvador.

## 2. Materials and Methods

### 2.1. Population Survery

A population-based cross-sectional survey was carried out in San Salvador (El Salvador). All data were collected during the period from April to May 2018. The sampling was made by convenience in 10 elementary schools (three private and seven public schools) that geographically cover four areas (South, East, Southwest, and downtown area) of the city of San Salvador. At least one group per grade in each school was included in the study (around 160 questionnaires per school). The questionnaires and informed consents were handed out to teachers whom attached them to the children’s homework notebooks. This process was carried out only once. If both the questionnaire and a signed informed consent were not returned after three working days, this was considered as non-response by the parents.

### 2.2. Questionnaire

A validated Spanish version of a structured questionnaire designed to estimate the parent-reported prevalence of FA in schoolchildren was used in this study [1,2]. The questionnaire takes into account strict criteria for defining FA and has high sensitivity to discriminate among IgE mediated FA and adverse food reactions. Furthermore, it can identify those children that at the time of the survey still had allergic reactions to the suspected food [2]. The questionnaire was culturally adapted, but the parameters to measure the variables of interest were not modified. Additionally, three questions about family history of atopy were included.

Respondents first answered questions related to basic demographic and clinical information about the child. All respondents with a positive response to perceived food-related recurrent symptoms completed the second part of the questionnaire. This section incorporated standardized questions about symptoms suggestive of IgE-mediated FA; time of appearance of the symptoms after food ingestion; the foods involved in the allergic/adverse food reaction; and treatments prescribed during allergic reactions among others. Also, all respondents answered three questions about the children’s family history of atopy. An Ethics Review Board of the School of Medicine of the Universidad Dr. José Matías Delgado reviewed the study protocol.

### 2.3. Definitions

Individuals were classified according to previously published definitions (Table 1) [1,2]. Convincing symptoms of immediate allergic reactions were: skin with hives, angio-edema, trouble breathing, wheezing or throat tightness, vomiting and diarrhea, among other symptoms typical of immediate hypersensitivity reactions that occurred within 2 h after food ingestion. The symptoms were considered to be recurrent if the parents stated that the symptoms were triggered every time that the children ingested the suspected food.

### 2.4. Statistical Analyses

Statistical analysis was carried out using PASW statistics version 22.0 (SPSS Inc., Chicago, IL, USA). Categorical variables were summarised by descriptive statistics including total numbers and percentages, and associations of FA with other atopic diseases, age, and season of birth were analysedby two-tailed Fisher exact test. Continuous variables were summarised by mean and range with differences between two groups calculated using the Student *t*-test. A *p*-value < 0.05 was considered statistically significant. Prevalence rates were calculated using OpenEpi software version 3.03a (www.OpenEpi.com, updated 06 April 2013 and accessed 05 May 2018). Rates were reported as rate (95% confidence intervals) per 100 inhabitants.

## 3. Results

### 3.1. Participants and Demographic Characteristics

Table 2 shows the demographic and clinical characteristics of the participants. A total of 1578 questionnaires were handed out. Of these, 508 were correctly answered (valid response rate, 32.19%). The other 979 were not returned (62.0%) or had invalid data (5.76%). The female/male ratio was 48.81/51.18 (*p* > 0.05). Allergic diseases were reported by 38.97% of the participants and 18.11% reported more than one allergic disease.

### 3.2. Parent-Reported Prevalence Rates of Adverse Food Reactions and FA

Prevalence estimations are show in the Table 3. Adverse food reactions were reported by 15.94% (*n* = 81) of the participants and more than 51.8% of these cases were perceived as allergic reactions. Except for the prevalence of physician-diagnosed FA, ever, the prevalence rates were higher in the 9–12 years old group than in the 4–8 one, but these differences were not statistically significant (*p* > 0.05) (Table 3). Twenty parents reported that their children had experienced typical symptoms of FA, but the symptoms occurred after 2 h of the ingestion of the suspected food and these cases were not considered for the prevalence estimations of immediate-type FA, either ever or current. Of these 20 cases, 11 parents reported that their children still had allergic reactions upon food exposure and were avoiding the suspected food from the children’s diets.

Having a family history of allergic disease was significantly associated with “immediate-type FA, ever” (60% vs. 40%) (*p* < 0.05). Similarly, asthma and rhinitis were more frequently reported in children with immediate-type FA, either “ever” or “current” (*n* = 45), than in children without convincing FA symptoms (*n* = 463) (*p* < 0.05). For all the variables evaluated, statistical comparisons by gender were not significant (*p* > 0.05).

**Figure 1:**
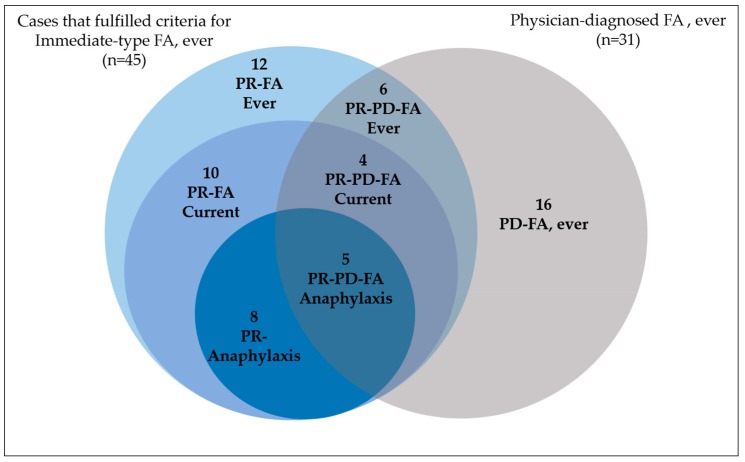
Characteristics of the cases that met criteria for immediate-type FA, ever, and/or PR-PD FA ever. Acronyms used: FA: Food allergy; PR: Parent-reported; PR-PD: Parent-reported physician-diagnosed.

PR-PD FA was reported by 33.3% (15 out of 45) of the “Immediate-type FA, ever” cases (Figure 1). Consequently, more than 50% of the PR-PD FA cases (*n* = 31) did not report convincing symptoms of “Immediate-type FA, ever” (Figure 1). Regarding anaphylaxis, only 5 (38.4%) out of 13 cases that fulfilled criteria for “food-dependent anaphylaxis” reported a physician diagnosis of FA. Most of the “food-dependent anaphylaxis” cases (60.5%) informed to have sought medical attention, but only 1 case reported the prescription of an epinephrine autoinjector. The parents of this anaphylactic case also reported that they did not buy the epinephrine device because in subsequent visits to the doctor the epinephrine autoinjector was not prescribed.

### 3.3. Foods Implicated in Adverse Food Reactions

The most commonly implicated foods causing recurrent adverse reactions were milk (6.1%) and chocolate (4.3%) followed by chili and shrimp (3.1% and 2.5% respectively). Skin with hives (49%), abdominal pain (37.0%), skin redness (33.3%) and swelling of lips/face (32.1%) were the most frequently reported symptoms (data not shown). Among those that reported adverse food reactions (*n* = 81), 55% (*n* = 45) sought medical attention.

### 3.4. Common Food Allergens and Clinical Characteristics of FA

**Figure 2:**
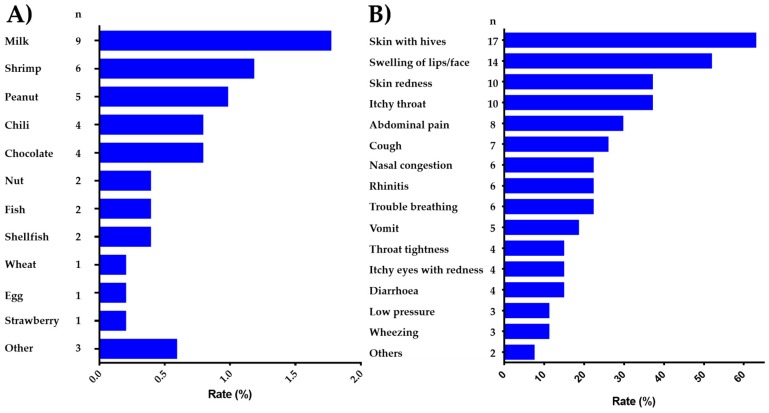
Specific food allergens and symptoms associated with “immediate-type FA, current”. (**A**) Prevalence of “immediate-type FA, current” by food in Salvadoran schoolchildren (*n* = 508); (**B**) Prevalence of specific symptoms in Salvadoran schoolchildren with “immediate-type FA, current” (*n* = 27).

The reported food allergens and the symptoms associated with food allergic reactions are show in Figure 2. The most commonly reported food allergens were milk (1.7%, 95% CI: 0.9–3.3), shrimp (1.3%, 95% CI: 0.6–2.8), peanut (0.98%, 95% CI: 0.4–2.2), chili (0.78%, 95% CI: 0.30–2.00) and chocolate (0.78%, 95% CI: 0.30–2.00) (Figure 2A). The most frequently reported specific symptoms were skin with hives (65.3%), swelling of lips/face (53.8%) and skin redness (38.4%) followed by itchy throat (38.4%) and abdominal pain (30.7%) (Figure 2B). Among the immediate-type FA, current, cases (*n* = 27), 51.8% (*n* = 14) received emergency medical attention. Of these last cases, 8 reported that were administered antihistamines to treat the symptoms. The adverse food reactions in children that fulfilled criteria for “immediate-type FA, current,” mainly affected the skin (85%) and the gastrointestinal (67%) and respiratory (52%) tracts. The main foods implicated were milk and shrimp (Figure 2B).

## 4. Discussion

The prevalence of food allergy in Salvadoran schoolchildren was estimated by parental-report in this study. The prevalence estimation is in line with similar studies carried out in European [9] and Asian [15,16] populations, but it is higher than that reported in other studies carried out in Latin American countries (1.6 to 1.8 times) such as Chile [1] and México [2]. Importantly, the Chilean and Mexican studies utilized the same instrument and included similar target populations. On the contrary, other studies carried out in Brazil [17] and Colombia [13] have reported lower prevalence rates (up to 2.6 times less), but the target populations differed. Whereas in this study the parents of schoolchildren were surveyed, in the Brazilian and Colombian studies the target populations were parents of infants/preschoolers and people ages 1–83 years, respectively. In contrast with studies carried out in high income countries, in this and other studies carried out in Latin American countries the prevalence rates of FA were higher in older than in younger children [1,2,13]. Although the prevalence rates were non-significant in all cases, this trend remains to be explored. A second deep questionnaire or interview could be helpful for such a purpose. Differences in FA prevalence rates among age-matched groups from different regions around the world can be attributed to cultural differences and feeding patters [14,18]. Furthermore, the genetic heritage and socioeconomic aspects, which vary in each country, could play important roles for triggering FA [14,19,20]. Therefore, the study of the epidemiology of FA is of particular interest in unexplored regions.

In this study the most frequently reported food allergens were milk, shrimp and peanut. In line with studies carried out in Chilean population [1], but contrary to what was reported in Mexican schoolchildren [2], milk was the leading food allergen reported by the parents of the Salvadoran schoolchildren. Regarding shrimp and other shellfish allergy, these are very common allergies either in Salvadoran or Mexican schoolchildren, but not in Chilean schoolchildren [1,2]. Overall, most food allergens reported by the parents of the Salvadoran schoolchildren match with those reported in the Mexican and Chilean studies, but the prevalence rates by specific foods substantially differ among the three studies. These findings support the notion that the relevance of the food allergens could differ among different regions [2].

Anaphylaxis is a “severe, life-threatening, generalized or systemic hypersensitivity reaction” [21]. In this study, gastrointestinal symptoms were less frequently reported than skin-related ones, trouble breathing, and low pressure, as previously reported [1,2,13,17,22]. Although the prevalence of food-induced anaphylaxis in Salvadoran schoolchildren was 2 times higher than that reported in Mexican ones [2], such a prevalence rate was similar to that reported in Chilean schoolchildren [1], using the same definitions of anaphylaxis. Certainly, the presence of allergic diseases other than FA has been associated with an increased vulnerability to anaphylaxis [2,7,14]. In line with this, most parents of the Salvadoran schoolchildren with “food-induced anaphylaxis” reported at least another atopic condition such as rhinitis, atopic dermatitis, insect sting allergy, and urticarial. These anaphylactic cases were mainly triggered after the exposure to milk and shrimp, similar to previous studies [2]. It should be noted that some species of chili could trigger symptoms such as red face, cough, and rhinitis, and these symptoms were reported in three chili allergy cases that met criteria for “food-induced anaphylaxis”. Similarly, strawberry could trigger allergic-like symptoms in some not sensitized individuals due to its histamine content [23,24].

Despite anaphylaxis was not uncommon among the Salvadoran schoolchildren that fulfilled criteria for “immediate-type FA, current”, most anaphylactic cases did not report the prescription of an epinephrine autoinjector. The lack of prescription of epinephrine devices has been previously reported in other Latin American studies [1,2]. Notably, the preferred mean for emergency treatment of anaphylaxis is the use of epinephrine autoinjectors [25]. These findings corroborate that food-induced anaphylaxis is not optimally managed in some Latin American countries and highlight the need to disseminate information on the risks of FA and treatment of acute food-induced allergic reactions among healthcare personnel [1,2]. Finally, we should highlight that there is a lack of anaphylaxis guides in most Latin American countries [26] and epinephrine autoinjectors are not easy to find in the mainstream drugstores of some Latin American cities [2,26].

The main strengths of our study are its population-based design, which include schoolchildren with different socioeconomic status, and the criteria used to estimate the prevalence rates of “immediate-type FA” and “food-induced anaphylaxis”. Notably, it has been reported that at least 93% of the subjects fulfilling these criteria had IgE antibodies to the implicated food [27]. However, we should acknowledge that our study has some limitations. First, the relatively low participation rate (32.19%) could influence the prevalence estimations. It is expected that people with atopic conditions will be more enthusiastic to take the survey than others. Secondly, detailed information about the medical diagnosis of FA was not collected in the parent-reported physician-diagnosed cases. And Thirdly, the immediate-type FA cases were not confirmed with objective diagnostic tests such as skin prick tests, specific IgEs, or oral food challenges. Despite these limitations, the present study is the first to report data about the prevalence, management, and clinical manifestations of FA in a Central American population and serves as the groundwork for further epidemiological studies based on objective diagnostic criteria.

## 5. Conclusions

To our knowledge, this is the first population-based study conducted in a Central American country to estimate the prevalence of FA. Overall, the data suggest that there is an increased prevalence of FA in Salvadoran schoolchildren compared to age-matched populations from other Latin American countries. Furthermore, the main food allergens triggering immediate-type allergic reactions could differ among the countries. Life-threatening anaphylaxis was reported by almost half of the immediate-type FA cases, but both a low prescription of epinephrine autoinjectors and a lack of their use by anaphylactic individuals were reported. Thus, actions should be taken to encourage the prescription and use of the autoinjectors in anaphylactic cases.

## Figures and Tables

**Table 1:** Definitions utilized in this study.

Condition	Criteria
Perceived FA, ever	The parents stated that their child has had allergic reactions to food.
Adverse food reaction	Any symptomatic recurrent adverse reaction to a specific food potentially mediated or not by immune mechanisms.
Immediate-type FA, ever	Having symptomatic recurrent adverse food reactions that were convincing of immediate-type hypersensitivity allergic reactions.
Immediate-type FA, current	Those cases that met criteria for “immediate-type FA, ever”, but answered negatively to the question “is your child now able to eat the suspected food(s) without any reactions”.
Food-dependent anaphylaxis	Those cases that met criteria for “immediate-type FA, current” and according to the three following criteria: (1) Acute onset of an illness with involvement of the skin, mucosal tissue or both and respiratory compromise or reduced blood pressure. (2) Two or more of the following that occur rapidly after food ingestion: (a) involvement of the skin-mucosal tissue, (b) respiratory compromise, (c) reduced blood pressure, (d) persistent gastrointestinal symptoms. (3) Reduced blood pressure after exposure to a food allergen.
Parent-reported physician-diagnosed (PR-PD) FA, ever	Those cases that answered positively to the question, “Has a doctor ever told you that your child has FA?”.

Acronyms used: FA: Food Allergy, PR-PD: Parent-reported physician-diagnosed.

**Table 2:** Demographic and clinical characteristics of the study population.

Variable	
Mean age in years (range)	9.2 (4–12)
Gender	*n* (%)
Female	248 (48.81)
Male	260 (51.18)
Known allergic diseases other than FA	
Allergic rhinitis	64 (12.59)
Atopic dermatitis	34 (6.69)
Insect sting allergy	84 (16.53)
Asthma	43 (8.46)
Urticaria	22 (4.33)
Drug allergy	37 (7.28)
Conjunctivitis	34 (6.69)
Anaphylaxis	1 (0.19)
Animals allergy	36 (7.08)

**Table 3:** Prevalence estimations.

Assessment	Number of Reported Cases	Prevalence % (95% CI)			*p*
		4–8 Years, *n* = 174	9–12 Years, *n* = 334	Total, *n* = 508	
Adverse food reactions	81	13.21 (8.97–19.05)	17.36 (13.68–21.79)	15.94 (13.02–19.38)	0.252
Perceived FA, ever	59	8.62 (5.29–13.73)	13.17 (9.96–17.22)	11.61 (9.11–14.69)	0.146
Physician-diagnosed FA, ever	31	7.47(4.41–12.36)	5.38 (3.43–8.35)	6.10 (4.33–8.5)	0.435
Immediate-type FA, ever	45	5.74 (3.15–10.25)	10.47 (7.63–14.22)	8.85 (6.68–11.65)	0.841
Immediate-type FA, current	27	3.44 (1.59–7.31)	6.28 (4.14–9.42)	5.31 (3.67–7.62)	0.214
Food-induced anaphylaxis	13	1.14 (0.31–4.09)	3.29 (1.84–5.8)	2.55 (1.50–4.32)	0.235

## References

[1] Hoyos-Bachiloglu R., Ivanovic-Zuvic D., Alvarez J., Linn K., Thöne N., de Los Ángeles Paul M., Borzutzky A. (2014). Prevalence of parent-reported immediate hypersensitivity food allergy in chilean school-aged children. Allergol. Immunopathol..

[2] Ontiveros N., Valdez-Meza E., Vergara-Jiménez M., Canizalez-Román A., Borzutzky A., Cabrera-Chávez F. (2016). Parent-reported prevalence of food allergy in mexican schoolchildren: A population-based study. Allergol. Immunopathol..

[3] DunnGalvin A., Dubois A., Flokstra-de Blok B., Hourihane J.O.B. (2015). The effects of food allergy on quality of life. Food Allergy: Molecular Basis and Clinical Practice.

[4] Gupta R., Holdford D., Bilaver L., Dyer A., Holl J.L., Meltzer D. (2013). The economic impact of childhood food allergy in the united states. JAMA Pediatr..

[5] Stensgaard A., Bindslev-Jensen C., Nielsen D., Munch M., DunnGalvin A. (2017). Quality of life in childhood, adolescence and adult food allergy: Patient and parent perspectives. Clin. Exp. Allergy.

[6] Hefle S.L., Furlong T.J., Niemann L., Lemon-Mule H., Sicherer S., Taylor S.L. (2007). Consumer attitudes and risks associated with packaged foods having advisory labeling regarding the presence of peanuts. J. Allergy Clin. Immunol..

[7] Prescott S.L., Pawankar R., Allen K.J., Campbell D.E., Sinn J.K., Fiocchi A., Ebisawa M., Sampson H.A., Beyer K., Lee B.-W. (2013). A global survey of changing patterns of food allergy burden in children. World Allergy Organ. J..

[8] Ontiveros N., Flores-Mendoza L., Canizalez-Román V., Cabrera-Chavez F. (2014). Food allergy: Prevalence and food technology approaches for the control of IgE-mediated food allergy. Austin J. Nutr. Food Sci..

[9] Rona R.J., Keil T., Summers C., Gislason D., Zuidmeer L., Sodergren E., Sigurdardottir S.T., Lindner T., Goldhahn K., Dahlstrom J. (2007). The prevalence of food allergy: A meta-analysis. J. Allergy Clin. Immunol..

[10] McBride D., Keil T., Grabenhenrich L., Dubakiene R., Drasutiene G., Fiocchi A., Dahdah L., Sprikkelman A., Schoemaker A., Roberts G. (2012). The europrevall birth cohort study on food allergy: Baseline characteristics of 12,000 newborns and their families from nine european countries. Pediatr. Allergy Immunol..

[11] McGowan E.C., Matsui E., McCormack M.C., Pollack C.E., Roger P., Keet C.A. (2015). The effect of poverty, urbanization, and race/ethnicity on perceived food allergy in the united states. Ann. Allergy Asthma Immunol..

[12] Soller L., Ben-Shoshan M., Harrington D.W., Knoll M., Fragapane J., Joseph L., Pierre Y.S., La Vieille S., Wilson K., Elliott S.J. (2015). Prevalence and predictors of food allergy in Canada: A focus on vulnerable populations. J. Allergy Clin. Immunol..

[13] Marrugo J., Hernández L., Villalba V. (2008). Prevalence of self-reported food allergy in Cartagena (Colombia) population. Allergol. Immunopathol..

[14] Guimarães T., Gonçalves L., Silva R., da Silva Segundo G.R. (2015). Prevalence of parent-reported food allergy in infants and preschoolers in brazil. Allergol. Immunopathol..

[15] Lee S.I., Shin M.H., Lee H.B., Lee J.S., Son B.K., Koh Y.Y., Kim K.E., Ahn Y.O. (2001). Prevalences of symptoms of asthma and other allergic diseases in Korean children: A nationwide questionnaire survey. J. Korean Med. Sci..

[16] Leung T.F., Yung E., Wong Y.S., Lam C.W., Wong G.W. (2009). Parent-reported adverse food reactions in Hong Kong Chinese pre-schoolers: Epidemiology, clinical spectrum and risk factors. Pediatr. Allergy Immunol..

[17] Gonçalves L., Guimarães T., Silva R., Cheik M., de Ramos Nápolis A., e Silva G.B., Segundo G. (2016). Prevalence of food allergy in infants and pre-schoolers in brazil. Allergol. Immunopathol..

[18] Woods R., Abramson M., Bailey M., Walters E. (2001). International prevalences of reported food allergies and intolerances. Comparisons arising from the European Community Respiratory Health Survey (ECRHS) 1991–1994. Eur. J. Clin. Nutr..

[19] Sicherer S.H. (2011). Epidemiology of food allergy. J. Allergy Clin. Immunol..

[20] Tosca M., Pistorio A., Accogli A., Rossi G.A., Ciprandi G. (2015). Food anaphylaxis in children: Peculiarity of characteristics. Allergol. Immunopathol..

[21] Panesar S., Javad S., De Silva D., Nwaru B., Hickstein L., Muraro A., Roberts G., Worm M., Bilò M., Cardona V. (2013). The epidemiology of anaphylaxis in Europe: A systematic review. Allergy.

[22] Wu T.C., Tsai T.C., Huang C.F., Chang F.Y., Lin C.C., Huang I.F., Chu C.H., Lau B.H., Wu L., Peng H.J. (2012). Prevalence of food allergy in Taiwan: A questionnaire-based survey. Intern. Med. J..

[23] Ibranji A., Nikolla E., Loloci G., Mingomataj E. (2015). A case report on transitory histamine intolerance from strawberry intake in a 15 month old child with acute gastroenteritis. Clin. Transl. Allergy.

[24] Kovacova-Hanuskova E., Buday T., Gavliakova S., Plevkova J. (2015). Histamine, histamine intoxication and intolerance. Allergol. Immunopathol..

[25] Simons F.E.R., Ardusso L.R., Bilò M.B., El-Gamal Y.M., Ledford D.K., Ring J., Sanchez-Borges M., Senna G.E., Sheikh A., Thong B.Y. (2011). World allergy organization guidelines for the assessment and management of anaphylaxis. World Allergy Organ. J..

[26] Cardona V., Álvarez-Perea A., Ansotegui I.J., Arias-Cruz A., González-Díaz S.N., Latour-Staffeld P., Ivancevich J.C., Sánchez-Borges M., Serrano C., Solé D. (2017). Management of anaphylaxis in Latin America: Current situation. Rev. Alerg. Mex..

[27] Sicherer S.H., Burks A.W., Sampson H.A. (1998). Clinical features of acute allergic reactions to peanut and tree nuts in children. Pediatrics.

